# 525 Evaluating Adherence to a New Pre-Operative Enteral Feeding Guideline in a Single Burn Center

**DOI:** 10.1093/jbcr/irae036.160

**Published:** 2024-04-17

**Authors:** Molly Marsh, Aryn Cruz, Lori Chrisco, Jamie L Hollowell, Ashley Levine, Rabia Nizamani, booker King, Felicia Williams

**Affiliations:** University of North Carolina, Chapel Hill, NC; UNC Hospital, Chapel Hill, NC; NC Jaycee Burn Center, Chapel Hill, NC; University of North Carolina, Durham, NC; University of North Carolina at Chapel Hill, Chapel Hill, NC; University of North Carolina, Chapel Hill, NC; UNC Hospital, Chapel Hill, NC; NC Jaycee Burn Center, Chapel Hill, NC; University of North Carolina, Durham, NC; University of North Carolina at Chapel Hill, Chapel Hill, NC; University of North Carolina, Chapel Hill, NC; UNC Hospital, Chapel Hill, NC; NC Jaycee Burn Center, Chapel Hill, NC; University of North Carolina, Durham, NC; University of North Carolina at Chapel Hill, Chapel Hill, NC; University of North Carolina, Chapel Hill, NC; UNC Hospital, Chapel Hill, NC; NC Jaycee Burn Center, Chapel Hill, NC; University of North Carolina, Durham, NC; University of North Carolina at Chapel Hill, Chapel Hill, NC; University of North Carolina, Chapel Hill, NC; UNC Hospital, Chapel Hill, NC; NC Jaycee Burn Center, Chapel Hill, NC; University of North Carolina, Durham, NC; University of North Carolina at Chapel Hill, Chapel Hill, NC; University of North Carolina, Chapel Hill, NC; UNC Hospital, Chapel Hill, NC; NC Jaycee Burn Center, Chapel Hill, NC; University of North Carolina, Durham, NC; University of North Carolina at Chapel Hill, Chapel Hill, NC; University of North Carolina, Chapel Hill, NC; UNC Hospital, Chapel Hill, NC; NC Jaycee Burn Center, Chapel Hill, NC; University of North Carolina, Durham, NC; University of North Carolina at Chapel Hill, Chapel Hill, NC; University of North Carolina, Chapel Hill, NC; UNC Hospital, Chapel Hill, NC; NC Jaycee Burn Center, Chapel Hill, NC; University of North Carolina, Durham, NC; University of North Carolina at Chapel Hill, Chapel Hill, NC

## Abstract

**Introduction:**

Malnutrition increases hospital length of stay, mortality, readmissions, overall costs, and also delays healing. Enteral nutrition via tube feeding (TF) is often used as primary or supplemental nutrition for burn patients. Our institution recently adopted a new policy to reduce TF hold times pre-operatively. An algorithm determining when TF are held pre-operatively to mitigate full body catabolism considers type of TF access (gastric or post-pyloric), the presence of a secure airway, and if the surgery involves the aerodigestive tract in order. We aimed to evaluate our compliance with the initiation of these new guidelines.

**Methods:**

This was a single center, retrospective study using our institution’s electronic medical record (EHR) admission list for patients on the burn service. Inclusion criteria consisted of patients admitted from June 1 through September 26, 2023, >18 years of age, had >20% TBSA burns requiring operative intervention, and received TF pre-operatively for at least one of their surgeries. Data collection included burn diagnosis, operating room (OR) date and time, feeding access, if the patient would be prone, receiving aerodigestive surgery or extubated post operative, NPO time ordered versus NPO time desired per guidelines, if the feeding protocol was followed, the hours of missed nutrition based on the expected feeding times outlined in the new policy, the calculated loss of protein and energy, and the percent of estimated nutrition needs lost.

**Results:**

There were 9 patients (n=9) who met the inclusion criteria and underwent a combined total of 14 surgeries. Out of the 14 operative days, the policy for the new NPO policy was only followed on one day (7.1%). These policy deviations led to an average of 6.7 missed hours of tube feed, 931 missed kilocalories, 60 grams of protein, 32.7% estimated energy needs, and 31.9% estimated protein needs per operative trip. All patients survived.

**Conclusions:**

Missed opportunities for enteral nutrition led to significant morbidity for our patients. Despite global acceptance to this guideline, compliance was suboptimal and resulted in substantial loss in nutrient delivery for our patients. Further education, time to allow culture change to take effect, and a standardized EHR order set can contribute to the success of this guideline in the future.

**Applicability of Research to Practice:**

This was one step of many to improve nutrient delivery to burn patients in our center and attenuate the risk and adverse effects of malnutrition for our patients. Reducing pauses in TF is a critical step in optimizing nutrient delivery for patients with high metabolic demands in order to improve outcomes.

